# Prescient diagnostic analysis for boron nutritional status in soy crops

**DOI:** 10.1038/s41598-022-26263-2

**Published:** 2023-02-09

**Authors:** Edilaine Istéfani Franklin Traspadini, Paulo Guilherme Salvador Wadt, Renato de Mello Prado, Cassiano Garcia Roque, Carlos Roberto Wassolowski

**Affiliations:** 1 Universidade Estadual Paulista, Faculdade de Ciências Agrárias e Veterinárias, Campus Jaboticabal, Via de Acesso Professor Paulo Donato Castelane, S/N-Vila Industrial, Jaboticabal, São Paulo 14884-900 Brasil; 2 Empresa Brasileira de Pesquisa Agropecuária, Embrapa-Rondônia, Rodovia BR 364 Km 5,5, Porto Velho, Rondônia 76815-800 Brasil; 3 Campus de Chapadão do Sul, Universidade Federal de Mato Grosso Do Sul, Rodovia MS-306, Km 105.-Zona Rural, Chapadão do Sul, Mato Grosso do Sul 79560-000 Brasil

**Keywords:** Plant reproduction, Environmental sciences

## Abstract

Boron is the most limiting micronutrient for soybean yield; therefore, accurate identification of its nutritional status is important for adequate fertilization management and maximize soybean yield potential. Currently, tools for nutritional status interpretation of B, such as the CND and DRIS are used; however, their efficacy is not considered to identify the true nutritional status of B. In this research, we investigated the efficacy of these methods to identify the nutritional status of B in 140 commercial soybean crops to obtain nutritional standards for the DRIS and CND methods. In addition, an experiment of B dose calibration (0, 300, 600, 1200 and 1800 g ha^−1^) was installed to assess the quality of nutritional diagnoses using the PDA. The experimental approach tested the limits of 0.25, 0.50, and 1.00 for the NRr and values of 1%, 5%, or 10% for YR. The DRIS method was more effective, and, on average, its variations increased yield by 27% compared to CND, with the best performance of DRIS when NRr = 1.00 was adopted with 10% for YR. This study highlights the need for reliable and accurate diagnostic methods with global implications for crop sustainability by improving the efficacy of B fertilization programs and crop yield.

## Introduction

In soybean, boron (B) fertilization is required to achieve high yields and good quality seeds^1, 2^, especially in acidic soils with low organic matter (OM) contents, where boron (B) is the most limiting micronutrient for high soybean yield^3^. Foliar spray is the most used technology for B fertilization in soybean crops^4–6^. This type of fertilization meets the soybean crop demand for B because the amounts are relatively low and, as B is a micronutrient, its absorption via foliar spray is relatively high^7^.

To achieve better foliar fertilization practices, numerous studies have been conducted on soybean crops^4, 8–13^ with emphasis on diagnostic methods based on nutritional balance, such as the Diagnosis and Recommendation Integrated system (DRIS) and the Compositional Nutrient Diagnosis (CND). In this way, it enabled divers advances have been achieved over the years, such as (a) selection of bivariate ratios to calculate DRIS indices by the Variance ratio (DrisVar)^14^; (b) the F-Test (DrisFtest)^15^; (c) adoption of direct and indirect relationships^16^; (d) use of the Logarithmic ratio transformation (DrisLog)^17^ (e) use of multivariate relationships^18^; and (f) nutrient responsiveness (NR) criterion^19^. It can be seen that there were many changes indicated for the calculation of diagnostic methods to increase the effectiveness of the diagnoses produced from different crops.

On the other hand, evaluation of the efficacy of these methods primarily compares the degree of agreement of the diagnoses produced with each other or by comparing the diagnoses with those from the conventional methods. Nevertheless, this type of assessment presents a significant distortion, as a high agreement degree could be obtained between criteria with low effectiveness to identify true deficiencies^20, 21^. Therefore, this form of nutritional assessment has significant scientific weakness, indicating that studies are needed for more efficient diagnostic methods.

This results in many of the agronomic research works, the assessment of the true nutritional status based on the positive response of crops to the correction of deficiencies has been neglected. Studies are restricted to a few cases, such as the works on soybean^22^, banana^23^, eucalyptus seedlings^24^, and sugarcane^20, 25^, which adopted the Prescient Diagnostic Analysis (PDA) recommended to assess the quality of nutritional diagnoses^26^. In soybean, different diagnostic methods has been adopted^8–13^ without it going to accuracy evaluation, except the studies that did show unsatisfactory results for the methods DRIS^22^ and CND^27^ to nutritional diagnosis of soybean..

The use of low-accuracy methods may produce inaccurate diagnoses, constituting a major limitation to optimize the use of fertilizers. The science of accurate nutritional diagnosis in crops should advance, due to the high cost of fertilizers and the finite nature of crop resources, which requires precise tools for the decision-making regarding fertilization to ensure economic return of the agricultural activity. This is even more relevant in crops like soy which is one of the main commodities in the world.

We propose that the hypothesis is that adjustments applied to a diagnostic method improves the quality of diagnoses. Therefore, possible adjustments to the DRIS and CND methods should improve their efficiency in predicting diagnoses for B status in soybean.

This research investigated the most effective method to diagnose the nutritional status of B in soybean, comparing the CND method with variations of the DRIS method, with the aim to identify the best tool for B diagnoses in soybean.

## Results

The nutrient responsiveness range (NRr) was tested ranging from 1.00 to 0.25. As NRr reduced, the frequency of cases of diagnosis of nutritional deficiency for B increased for all diagnostic methods tested. This increase in the deficiency frequency of cases was lower for the DrisVar and DrisFtest methods, intermediate for CND and DrisLog methods, and higher for the DrisAll method (Table 1). The DrisAll was the most sensitive method to changes in the NRr value, ranging from 0 to 25% of cases of B deficiency, while the DrisVar and DrisFtest were the least sensitive to changes in NRr (Table 1).

**Table 1:** Percentage of deficiency diagnosis interpreted by the DRIS and CND methods, as a function of the nutrient responsiveness range (NRr) for B diagnosis in soybean, cultivated in Chapadão do Sul, Mato Grosso do Sul, Brazil.

Methods^a^	NRr (%)		
	1.0	0.50	0.25
DrisAll	0	5	25
DrisFtest	0	0	5
DrisVar	0	5	5
DrisLog	0	5	20
CND	5	15	20

^a^Methods DRIS adopting the criteria of selecting all bivariate relations—DrisAll, using the criterion Ratio of Variances—DrisVar, using the F test—DrisFtest and using the logarithmic transformation—DrisLog and compositional nutrient diagnosis—CND.

The difference between the various diagnostic methods was significant for low NRr values. For NRr = 1.00 only the CND method differed from the others, while NRr = 0.25 showed the greatest difference in the capacity of the methods to identify cases of B deficiency.

Applying the comparison between the diagnoses for the entire set of soybean crops, except for the DrisLog method, all the other methods presented Parity Grade (PG) above 90%, with NRr = 1.00 (Table 2). The main trend was the PG reduction as the NRr also reduced. The biggest difference for PG was 21%, when NRr ranged from 1.00 to 0.25 for the comparison between the DrisFtest and DrisVar methods. The smallest difference was 1% in the PG when the NRr ranged from 1.00 to 0.25 in the comparison between the CND and DrisVar methods (Table 2).

**Table 2:** Parity grade (PG) obtained between the diagnoses of the DRIS and CND methods adopting different range factors adopted in the nutrient responsiveness range (NRr) for the diagnosis of B in soybean, cultivated in Chapadão do Sul, Mato Grosso do Sul, Brazil.

Degree of agreement (%)						
		DrisAll	DrisFtest	DrisVar	DrisLog	CND
DrisAll	1.00	–				
	0.50	–				
	0.25	–				
DrisFtest	1.00	93	–			
	0.50	81	–			
	0.25	75	–			
DrisVar	1.00	95	96	–		
	0.50	85	88	–		
	0.25	75	88	–		
DrisLog	1.00	85	84	83	–	
	0.50	72	64	72	–	
	0.25	79	63	70	–	
CND	1.00	91	95	92	84	–
	0.50	79	85	84	69	–
	0.25	79	84	91	68	–

DRIS method adopting the criteria of selecting all bivariate relations—DrisAll, using the criterion Ratio of Variances—DrisVar, using the F test—DrisFtest and using the logarithmic transformation—DrisLog and compositional nutrient diagnosis—CND.

Methods with similar results regarding the ability to indicate B-deficient soybean crops showed different behavior when compared to PG, such as in the comparison between CND and DrisLog or between DrisFtest and DrisVar. On the other hand, different methods showed higher PG regarding the capacity to indicate B-deficient soybean crops, such as the comparison between DrisAll and DrisVar (Table 2).

**Figure 1:**
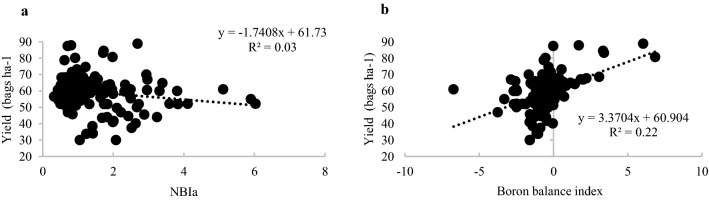
Relationship between soybean yield and the Average Nutrient Balance Index (NBIa) (**a**) and the boron balance index (**b**) obtained by the DRIS method using all bivariate relationships (DrisAll) for the sampled dataset for soybean, cultivated in Chapadão do Sul, Mato Grosso do Sul, Brazil.

**Figure 2:**
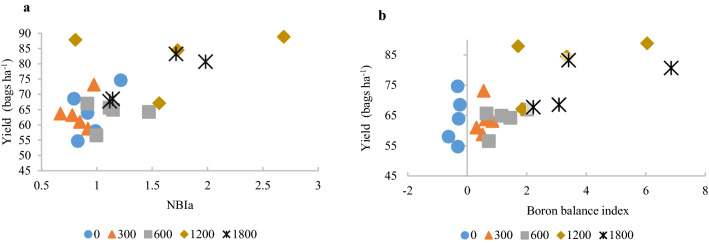
Relationship between soybean yield and the Average Nutrient Balance Index (NBIa) (**a**) and the boron balance index (**b**) obtained by the DRIS method using all bivariate relationships (DrisAll) in the dataset of the calibration experiment of B in soybean (doses 0, 300, 600, 1200, and 1800 kg ha^−1^ of B), cultivated in Chapadão do Sul, Mato Grosso do Sul, Brazil.

The dispersion of yield values was higher for low Average Nutrient Balance Index—NBIa values (Figs. 1a and 2a) or for boron balance index—B-index close to zero (Figs. 1b and 2b), for all soybean crops (Fig. 1), or experimental crops (Fig. 2). For higher NBIa values, or B-index far from zero, the yield values of soybean crops showed lower dispersion and were equal to or above the average of the data set evaluated (Figs. 1 and 2).

The B-index values for crops without B application (0 kg ha^−1^ of B) were negative (Fig. 2b). The B application showed positive B-index values, tending towards nutritional excess at the highest doses (1200 to 1800 kg ha^−1^ of B) (Fig. 2b). The same effect was verified for the NBIa x yield ratio: when increasing the applied B dose, higher values were obtained for NBIa (Fig. 2a).

The Accuracy for Deficiency—AccDef and Deficiency Ratio—DR indicators diverged between the diagnostic methods as the NRr was reduced from 1.00 to 0.25 and the net Yield Responses—YR from 10 to 1%; however, this divergence between the methods was practically annulled when the YR of 10% and NRr of 1.00 (Table 3).

**Table 3:** Percentage of identified cases of diagnoses true deficiency (TDef), false deficiency (FDef), true sufficiency (TSuf), false sufficiency (FSuf); accuracy (Acc); deficiency ratio (DR), sufficiency ratio (SR) accuracy for deficiency (AccDef), accuracy for sufficiency (AccSuf) obtained by the diagnosis of the DRIS and CND methods as a function of the nutrient responsiveness range (NRr) and yield response (YR) of the nutritional status of foliar boron in a soybean cultivar, in Chapadão do Sul, Mato Grosso do Sul, Brazil.

Method	NRr	YR (%)	VDef (%)	FDef (%)	VSuf (%)	FDef (%)	Acc (%)	AccDef (%)	AccSuf (%)	DR	SR
DrisAll	1.00	1	0	0	40	60	40	0	100	0.0	0.7
		5	0	0	55	45	55	0	100	0.0	1.2
		10	0	0	80	20	80	0	100	0.0	4.0
	0.5	1	5	0	40	55	45	8	100	1.0	0.7
		5	5	0	55	40	60	11	100	1.0	1.4
		10	0	5	75	20	75	0	94	0.0	3.7
	0.25	1	15	10	30	45	45	25	75	1.5	0.7
		5	15	10	45	30	60	33	82	1.5	1.5
		10	0	25	55	20	55	0	69	0.0	2.7
DrisVar	1.00	1	0	0	40	60	40	0	100	0.0	0.7
		5	0	0	55	45	55	0	100	0.0	1.2
		10	0	0	80	20	80	0	100	0.0	4.0
	0.5	1	5	0	40	55	45	8	100	1.0	0.7
		5	5	0	55	40	60	11	100	1.0	1.4
		10	0	5	75	20	75	0	94	0.0	3.7
	0.25	1	5	0	40	55	45	8	100	1.0	0.73
		5	5	0	55	40	60	11	100	1.0	1.4
		10	0	5	75	20	75	0	94	0.0	3.7
DrisFtest	1.00	1	0	0	40	60	40	0	100	0.0	0.7
		5	0	0	55	45	55	0	100	0.0	1.2
		10	0	0	80	20	80	0	100	0.0	4.0
	0.5	1	0	0	40	60	40	0	100	0.0	0.7
		5	0	0	55	45	55	0	100	0.0	1.2
		10	0	0	80	20	80	0	100	0.0	4.0
	0.25	1	5	0	40	55	45	8	100	1.0	0.7
		5	5	0	55	40	60	11	100	1.0	1.4
		10	0	5	75	20	75	0	94	0.0	3.7
DrisLog	1.00	1	0	0	40	60	40	0	100	0.0	0.7
		5	0	0	55	45	55	0	100	0.0	1.2
		10	0	0	80	20	80	0	100	0.0	4.0
	0.5	1	5	0	40	55	45	8	100	1.0	0.7
		5	5	0	55	40	60	11	100	1.0	1.4
		10	0	5	75	20	75	0	94	0.0	3.7
	0.25	1	10	10	30	50	40	17	75	1.0	0.6
		5	10	10	45	35	55	22	82	1.0	1.3
		10	0	20	60	20	60	0	75	0.0	3.0
CND	1.00	1	0	0	37	63	37	0	100	0.0	0.6
		5	0	0	53	47	53	0	100	0.0	1.1
		10	0	0	79	21	79	0	100	0.0	3.7
	0.5	1	5	5	32	58	37	8	86	1.0	0.5
		5	5	5	47	42	53	11	90	1.0	1.1
		10	5	5	74	16	79	25	93	1.0	4.6
	0.25	1	11	5	32	53	42	17	86	2.0	0.6
		5	11	5	47	37	58	22	90	2.0	1.2
		10	5	11	68	16	74	25	87	0.5	4.3

DRIS method adopting the criteria of selecting all bivariate relations—DrisAll, using the criterion Ratio of Variances—DrisVar, using the F test—DrisFtest and using the logarithmic transformation—DrisLog and compositional nutrient diagnosis—CND.

The Accuracy for Sufficiency—AccSuf and Sufficiency Radius—SR indicators differed slightly between the diagnostic methods with the best performance for these indicators achieved with YR of 10% and NRr of 1.00 (Table 3).

The CND method was the most efficient to identify True Deficiency—TDef cases, however it was also associated to the lowest number of False Sufficiency—Fsuf, resulting in higher rates for the DR indicator and better AccDef, mainly for NRr of 0.25 and YR of 1%. The DRIS methods showed a better performance for DR and AccDef measurements with NRr of 0.25 and YR of 5% (Table 3).

**Figure 3:**
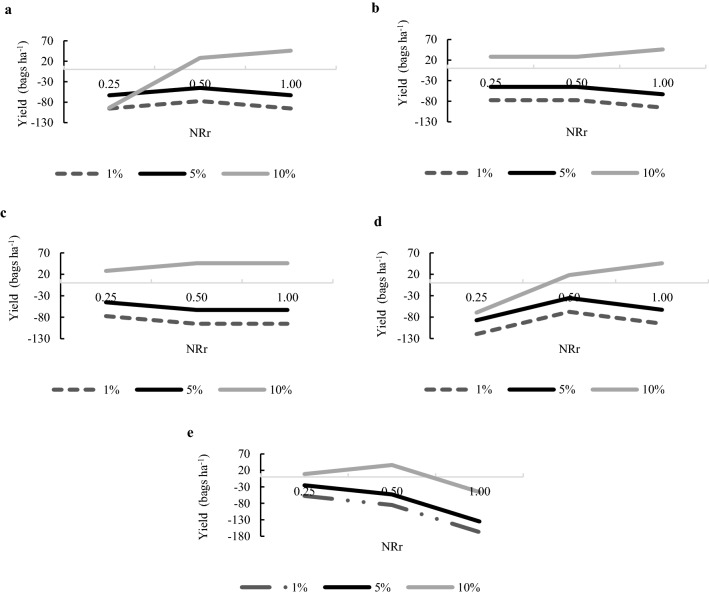
Net yield response [Net d(Y)] as a function of the nutritional diagnosis of B obtained by the DRIS method adopting the criteria of selecting all bivariate relationships—DrisAll (**a**), using the criterion Ratio of Variances—DrisVar (**b**), using the F test—DrisFtest (**c**) or using the logarithmic transformation—DrisLog (**d**) and compositional nutrient diagnosis—CND (**e**). using 0.25; 0.50 and 1.0 for nutrient responsiveness range (NRr) and 1%, 5% and 10% for yield response (YR).

The net yield response (Net d(Y)) was negative for NRr of 1.00 and YR = 1% or 5%. The CND method showed positive Net d(Y) only with YR of 10% and NRr of 0.50 (Fig. 3). Most variations of the DRIS method showed positive Net d(Y) with YR = 10%, except for DrisAll and DrisLog when NBr was 0.25 (Fig. 3).

Taking the Net d(Y) indicator as a reference for comparing the diagnostic methods, variations in the DRIS method resulted, on average, in a Net d(Y) 27% higher than that achieved by the CND (Fig. 3).

## Discussion

The diagnoses obtained by the DRIS and CND methods for B diagnosis in soybean presented a medium-to-high PG (68 to 95%). It was also observed a high PG (90%) for the DRIS and CND in the nutritional B diagnosis in soybean^10^. However, a high PG does not ensure that the interpretation of nutritional status is correct, as it only indicates that the interpretation methods are producing coincident or the same diagnoses^20, 28^.

In sugarcane, a higher PG did not always correspond to higher yield gains using nutritional diagnosis^20, 25^. But, in this study, the methods with highest PG were the same that obtained the best yield dispersion data, based on the NBIa and B-index, for the set of crops monitored (Fig. 1) or just for the experimental crops were not good indicators to evaluate the performance of the diagnostic method, as exemplified for the DrisAll method. This may be explained because, the theoretical model predicted for this dispersion consists of a greater range of yield values for low NBIa values and a smaller range of yield values for high NBIa values^29^, which was not observed for commercial or experimental crops.

Similar results were found in a study conducted under experimental conditions, with P application in sugarcane, reporting the occurrence of crops of medium and high yields in situation of plants with greater nutritional imbalance^20^. This may be associated to the premise that the nutrient alone is not the only determining factor in plant yield and that other factors, nutritional or not, may be the precursors of yield gains^7^.

## Analysis of diagnosis prediction

The adoption of a higher YR threshold value reduced the probability of the crop to be considered as having a true B deficiency. It was also found that a lower frequency of sugarcane plants in a state of nutritional deficiency for P, as the YR value ranged from 5 to 40%^20^.

The threshold defined for YR directly affected the accuracy indicators. Regardless of the diagnostic method evaluated, the best performance was observed when the limit of 10% was adopted for YR. Conversely, to sugarcane, higher efficiency of diagnosis by the CND method was attributed to the YR values of 35% and 40%^20^. This difference is explained by the greater responsiveness of nutrient P in sugarcane growth with yield gains between 30 and 35%^30, 31^. On the other hand, for B application in soybean, the plant response varied from null^31, 32^ to a maximum of 8% to 17%^1, 2^.

The DR indicator was ineffective to evaluate the performance of B diagnostic methods in soybean, as DR above 1.00, suggested as adequate^22^, resulted in a lower net yield response. It was also observed a low RD to evaluate the CND method, adopting NRr = 0.00, even though this was the diagnostic method with the best performance to assess the nutritional P status in sugarcane^20^. To eucalyptus seedlings the highest RD proportions were achieved by the most efficient method in the nutritional diagnosis, which varied according to the nutrient evaluated^24^.

Similarly, the CND method showed low efficiency to identify sugarcane nutritional status^20^. According to the authors, the lower efficiency of the CND can be explained by the low sensitivity of the method in making fine adjustments to the nutritional status or by the low assertiveness when greater yield gains are desired, given that positive net yield responses were observed only for YR above 20–30% and NRr = 0.00^20^.

The interpretation of B nutritional status by method CND did not show the minimum efficiency required to be recommended for the management of B fertilization in soybean, since it resulted in negative values for yield^27^. Nonetheless, after the adjustment on methodology proposed on this work (YR of 10% and NRr of 0.50), the method CND did show promising results to the diagnosis of B nutrition status, obtained positive Net d(Y) of de 40 bags ha^−1^.

For the DRIS method, the limit of NRr = 1.00 resulted in a better performance to identify the true B nutritional status in soybean. On the other hand, the limit of NRr = 0.25 showed a larger number of cases of false deficiency, compromising the primary objective of nutritional diagnosis to correctly identify nutritional deficiencies^22^. In this study, the better performance of DRIS (especially when using YR = 10% and NRr = 1.00) was mostly attributed to the greater sensitivity of the method to correctly identify cases of nutritional sufficiency, which may be a consequence of the lower responsiveness of soybean to boron fertilization^32, 33^.

In general, for a less responsive nutrient such as B, a lower value for NRr (aiming to increase the frequency of nutritional deficiency diagnoses) proved to be ineffective to identify the soybean True Nutritional Status—TNS of B. Unsatisfactory results for the nutritional diagnosis of N, P, and K in soybean were also related by other authors^22^. As the author obtained low accuracy, they adopted NRr = 0 (every nutrient with a negative DRIS index was considered deficient). Conversely**,** in a study on sugarcane with a responsive nutrient, such as P, Silva et al.^20^ concluded that increasing the probability of P deficiency responses (NRr = 0) resulted in better YR.

As the DRIS has several variations in its application, these results point to the need to perform an accuracy test to improve nutritional diagnosis. For example, DrisFTest was significant for presenting the highest yield in two different data adjustments (NRr = 0.50 with 10% for YR and, NRr = 1.00 with 10% for YR), even with a smaller increment of cases of deficiency diagnoses.

Our hypothesis that the adjustments made in the diagnostic procedures were effective. The methodological variations introduced in the DRIS method not only provided better accuracy to this method than that obtained by the CND method, as the variations applied to the diagnostic process also affected the quality of the diagnostics produced.

## Methods

We carried out assays of foliar B and nutritional monitoring of commercial soybean crops in experimental and commercial production sites, respectively, in the municipality of Chapadão do Sul, Mato Grosso do Sul State, Brazil. The dystrophic Red Latosol predominates in the experimental site and in most of the municipality^34^. The climate in the region is humid tropical (Aw) (Köppen classification) with a two-month dry season and an average annual rainfall of 1550 mm^35^.

All sites were monitored for nutritional status in the 2015/2016 harvest of commercial crops, in a no-tillage system in straw, but with different phytotechnical managements. We used cultivars with a determinate cycle: P98Y30, M8210 IPRO, M9144 RR, SYN1288 IPRO, BG4184, and 98Y52, indeterminate cycle: DESAFIO, W 791 RR, GMX CANCHEIRO RR, NS7670, and semi-determinate cycle: M7739, TEC7849 IPRO, M 7339 IPRO, AS3797 IPRO. The cultivars are recorded and can consulted in Registro Nacional de Cultivares—RNC^36^.

In each site, a perimeter of one hectare was delimited for leaf sampling. Yield was estimated in kg per hectare of soybean grain, adjusted for water content in the grain at 13%. We were having licenses to collect soy crop. For the experimental area, one foliar B fertilization test (calibration test) was designed to determine the B response curve on crop yield as well as leaf B levels in soybean cultivar DESAFIO. DESAFIO cultivar presents excellent productivity, and is highly responsive to nutrient availability. Having as phenotypeprofile the following characteristics: plant height of 80 cm, indeterminate growth habit, flowering of 35 days and with resistance to lodging^36^.

The complete attributes of soil fertility analysis in the experimental area of Fundação Chapadão, can be seen in a previously published study^27^. The soil of the experimental site presented 0.32 mg B dm^−3^ with extraction by hot water, a content interpreted as low^37^. The experimental design was randomized blocks, with five B doses (0, 300, 600, 1200, and 1800 g ha^−1^), that corresponded to 0%, 16%, 33%, 67%, and 100% of the recommended rate for soybean in the Cerrado (brazilian savannah) as B amendment^37^ applied as boric acid with five replicates per treatment.

In pre-sowing, 100 kg ha^−1^ of KCl was applied like broadcast application. At sowing, on Nov 24, 2015, we applied 115 kg ha^−1^ of monoammonium phosphate (N: 11% and P_2_O_5_: 52%). The total experimental site comprised 852.5 m^2^, divided into 25 experimental plots of 11.0 m long and 3.1 m wide. Each experimental plot consisted of seven planting rows, where the three central lines were the useful area for the evaluations with eight central meters used per row. In the soybean plant, boron was applied via foliar with a CO_2_ pump sprayer, adjusted to a spray volume of 150 L ha^−1^. To increase B absorption, 0.15% surfactant (nonionic polyoxymethylene surfactant) and 1% urea were mixed with the syrup. Each dose was divided into three applications, two in the vegetative phase (V2 and V5) and one at the beginning of flowering (R1). The applications were carried out in the morning at a temperature near 25 °C, relative humidity of about 80%, and wind speed near 7 km h^−1^.

After, soybean leaves were sampled in the experimental site at stage R1 (beginning of plant flowering) at 10 days after the last B application. Each sampling site was represented by the random collection of 25 fully expanded leaves, from the third trifoliate, with petiole, counted from the plant apex^38^.

The leaves sampled in the calibration test plots and in the commercial crops, containing the petiole, were washed in deionized water, in a detergent solution (0.1% v/v), and then rinsed with hydrochloric acid solution (0. 3% v/v) and deionized water. Subsequently, the leaves were dried in a forced circulation oven at 60–70 °C until reaching constant mass and then ground in a mill. Next, the nutrient contents were analyzed to different digestion processes^39^: microwave (K), sulfuric (N) and nitroperchloric (P, S, Ca, Mg, Mn, Fe, Zn, and Cu). After digestion, the leaf samples were analyzed to determine the contents of S, Ca, Mg, Mn, Fe, Zn, Cu (ICP-OES plasma spectrometry), K (flame photometry), P (molecular spectrophotometry) and B was extracted by combustion and determined by spectrometry. Total N was determined by Kjehdahl distillation. For each nutrient, we identified leaf samples with nutrient contents within ± 95% range of the mean in the 165 samples data set (was used data set of the soybean yield and B contents in experimental plots and commercial crops).

After sampling soybean leaves, when the plants was reached full maturation was to do mechanized harvesting of in commercial crops farms. In the experimental site, it took place on 05 April 2016, obtaining the productivity (Bags ha^−1^) of the plots. The bag is equivalent to 60 kg of grain. We were having licenses to collect soy crop.

The mathematical process for calculating the intermediate functions and to generate the nutritional indices for the DRIS were calculated following the formula originally^29^ and with logarithmic transformation of the relationships between nutrients (DRIS Logarithmic ratio—DrisLog)^17^.

For both process to calculate the nutrient balance index by the DRIS method, the F-Test criterion^15^ was used to select the direct and inverse relationships between the nutrients to compose the calculation of the DRIS indices, called the “DRIS F-Test (DrisFtest)”. For the first case, when there was no data transformation^29^, the variance ratio criterion^14^ was also used, here called the “DRIS Variance ratio (DrisVar)”. The use of all nutritional ratios was an alternative tested, without any selection^16^, called “DRIS All ratios (DrisAll)”.

To apply the DrisFtest and DrisVar criteria, the F value was calculated by the ratio between the variance (S^2^) of subpopulations from low to high yield for the direct and inverse nutrients relationships. The F tabulated was obtained based on the degrees of freedom by the number of crops in the low- and high-yield population minus one, at a significance level of 5%. Samples with yield above the mean + 0.25 of standard deviation were considered high-yielding populations.

For the CND method, was just used foliar samples that contained the 11 nutrients to obtain CND nutritional standards (norms), determining the means of the multivariate relationships of high yield^18^. After obtaining the CND norms, the nutrient balance index was obtained^10^. To interpret the nutrient balance index obtained by the DRIS and CND methods was adopted the criterion of the nutrient responsiveness method (NR)^19^, grouping the B-index into two classes: insufficient when the B-index was negative and, in module, above than the value of the “f × NBIa” (nutrient responsiveness range—NRr) (Eq. 1). In all other cases, the B-index was considered balanced. The values of 0.25, 0.50, and 1.00 for f were used.

1 $${\text{Insufficient}}:{\text{B-index}} < \, 0{\text{ and }}\left[ {{\text{B-index}} } \right] \, > {\text{ f }} { \times }{\text{ NBIa}}$$

We carried out the nutritional diagnosis of the experimental plots using the DRIS and CND methods, classifying the plots as B deficient or B sufficient. The accuracy of correctness of these diagnoses was performed by the APD procedure^26^ by comparing the diagnosis given by the diagnosis method (DRIS and CND) with the TNS.

The TNS is obtained by analyzing the soybean yield response in each experimental plot as a function of B application. Comparing a plot with B application with another without B application or with B applied at smaller doses. When the YR was of at least 1%, 5%, or 10% in soybean yield showed that the plot was truly B deficient/insufficient. In all other cases, the plot was truly adequate/balanced.

The deficiency diagnosis of the methods (DRIS and CND) was considered true (TDef) when there was a yield increase with the nutrient addition and false (FDef) when there was no yield increase. The sufficiency diagnosis of the methods (DRIS and CND) was considered true (TSuf) if B application did not increase yield and false (FSuf) if there was a yield increase (Table 4)^26^.

**Table 4:** Diagnosis of the nutritional status obtained by the interpretation method and the physiological true nutritional status (TNS) of the crop.

Number of cases		
Nutritional status interpretation	True nutritional status (TNS)	
	Responsive	Non responsive
Deficient	TDef	FDef
Sufficient	FSuf	TSuf
Subtotais	∑Def	∑Suf

*TDef* true deficiency, *FDef* false deficiency, *FSuf* false sufficiency, *TSuf* true sufficiency, *∑Def* sum of deficiency, *∑Suf* sum of sufficiency.

The quality of predictions was then quantified by different indicators, namely accuracy (Acc), net yield response (Net d(Y))^26^, deficiency ratio (DR), accuracy for deficiency (AccDef), and accuracy for sufficiency (AccSuf)^22^. A new calculation was introduced for the accuracy test, which is the ratio of the sufficiency radius (SR). The diagnostic quality indicators were created according to the number of counts of indicated categories^22, 26^.

The Acc is given by the percentage sum the of true diagnosis cases and obtained by the “Eq. (2)”, where n is the total number of comparisons performed.

2 $${\text{Acc }} = { 1}00* \, \left( {{\text{TDef}}/{\text{n }} + {\text{ TSuf}}/{\text{n}}} \right)$$

AccDef and AccSuf correspond to the percentage of correct answers in relation to the total of deficiency or sufficiency diagnoses, respectively, and were obtained by the “Eqs. (3) and (4)”, where VDef and ∑Suf are the sum of cases of deficiency and sufficiency, respectively.

3 $${\text{AccDef }} = { 1}00 \, { \times }{\text{ TDef }}/ \, \sum {\text{Def}}$$

4 $${\text{AccSuf }} = { 1}00 \, { \times }{\text{ TSuf}}/ \, \sum {\text{Suf}}$$

The DR was calculated by the ratio of true deficiency for false deficiency diagnoses and the SR was calculated by the ratio of true sufficiency for false sufficiency diagnoses, they were obtained by the “Eq. (5) and (6)”, respectively.

5 $${\text{RD}} = \% {\text{TDef}}/\% {\text{FDef}}$$

6 $${\text{SR}} = \% {\text{TSuf}}/\% {\text{FSuf}}$$

The Net d(Y) was obtained by the “Eq. (7)”. In this equation, the mathematical operation consists of adding or subtracting the productivity module of each plot, depending on whether the diagnosis is considered true (|P_VDEF| or |P_VSUF|) or false (|P_FDEF| or |P_FSUF|), for each state nutritional deficiency/insufficiency or sufficiency/balanced.

7 $${\text{Net d}}\left( {\text{Y}} \right) \, = \, \left| {{\text{P}}\_{\text{VDEF}}} \right| \, + \, \left| {{\text{P}}\_{\text{VSUF}}} \right| \, - \, \left| {{\text{P}}\_{\text{FDEF}}} \right| \, - \, \left| {{\text{P}}\_{\text{FSUF}}} \right|$$

The PG (Parity Grade) was also measured between the different diagnoses of the nutritional status of B provided by each of the diagnostic methods used. Between these methods, the true nutritional status was determined experimentally. The PG was calculated by the frequency of cases with the same diagnoses (in agreement between the methods) in relation to the total of diagnoses compared with each other.

The normal distribution of the soybean yield and B experimental plot data set was tested by the Shapiro–Wilk test. The analysis was performed with the AgroEstat statistical software^40^.

This research was not conducted with endangered species and all methods in this study were carried out in compliance/accordance with relevant institutional, national, and international guidelines and legislation.

## Conclusions

The DRIS method was more promising than the CND method to diagnose the B nutritional status in soybean, and it was also more sensitive to the nutrient responsiveness and to the YR value adjustment.

## Data Availability

The datasets generated during and/or analyzed during the current study are available from the corresponding author on reasonable request.
